# The joint Orthoplastic management of an Achilles tendon avulsion: A rare case report

**DOI:** 10.1016/j.jpra.2022.08.005

**Published:** 2022-09-02

**Authors:** S. Shah, A.S. Sehmbi, L. Jeyaseelan

**Affiliations:** a Queen's Hospital, Barking Havering Redbridge NHS trust, London, United Kingdom; b Royal London Hospital, Barts Health NHS trust, London, United Kingdom

**Keywords:** Achilles tendon avulsion, Achilles tendon rupture, Rotational flap, FHL tendon transfer, Primary repair

## Abstract

Rupture of the Achilles tendon typically occurs at the mid-substance, and less commonly at the distal insertion or proximal musculotendinous junction. We report the case of a 60-year-old multi-morbid patient presenting with an avulsion of the Achilles tendon from the gastrocnemius-soleus complex - a variant of injury previously unrecorded in the literature. Initial Orthoplastic management involved debridement and primary fixation of the avulsed tendon to the muscle with a concurrent lateral rotational flap. Flap failure and loss of tendon viability necessitated further debridement and eventual split-skin grafting (SSG). A residual dorsiflexion deformity will undoubtedly require further operative intervention. Here, we report the management of this unreported variant of Achilles tendon injury and discuss alternatives to our initial management that could have resulted in fewer procedures and improved long-term functional outcomes.

## Introduction

Achilles tendon rupture (ATR) has an annual incidence of 18 per 100,000 annually.^1^ There is a predilection for intermittently active middle-aged men with a with a mean reported age of between 37 - 44 years.^1^ The tendon plays a vital role in ambulation, accounting for approximately 93% of plantar flexion force.^1^ ATR is classically observed following a single high-load impact such a forced dorsiflexion preceding a jump or following a landing.

10% of cases report prodromal tendinopathy and established risk factors including steroid injection, fluroquinolone use and seronegative spondyloarthropathies.^2^ Rupture typically occurs in the vascular watershed segment three to six centimetres proximal to the calcaneal insertion^1^ and less commonly at the musculotendinous junction and calcaneal insertion.^3^

We present a case of Achilles tendon injury proximally, with a complete avulsion of the tendon from the gastrocnemius-soleus complex. To the best of our knowledge, this is the first report of this in the literature.

## Case Presentation

A 60-year-old male was presented to our level 1, major trauma centre, with an open injury to the posterior aspect of the left heel. following an accidental fall on a tugboat.

**Fig. 1:**
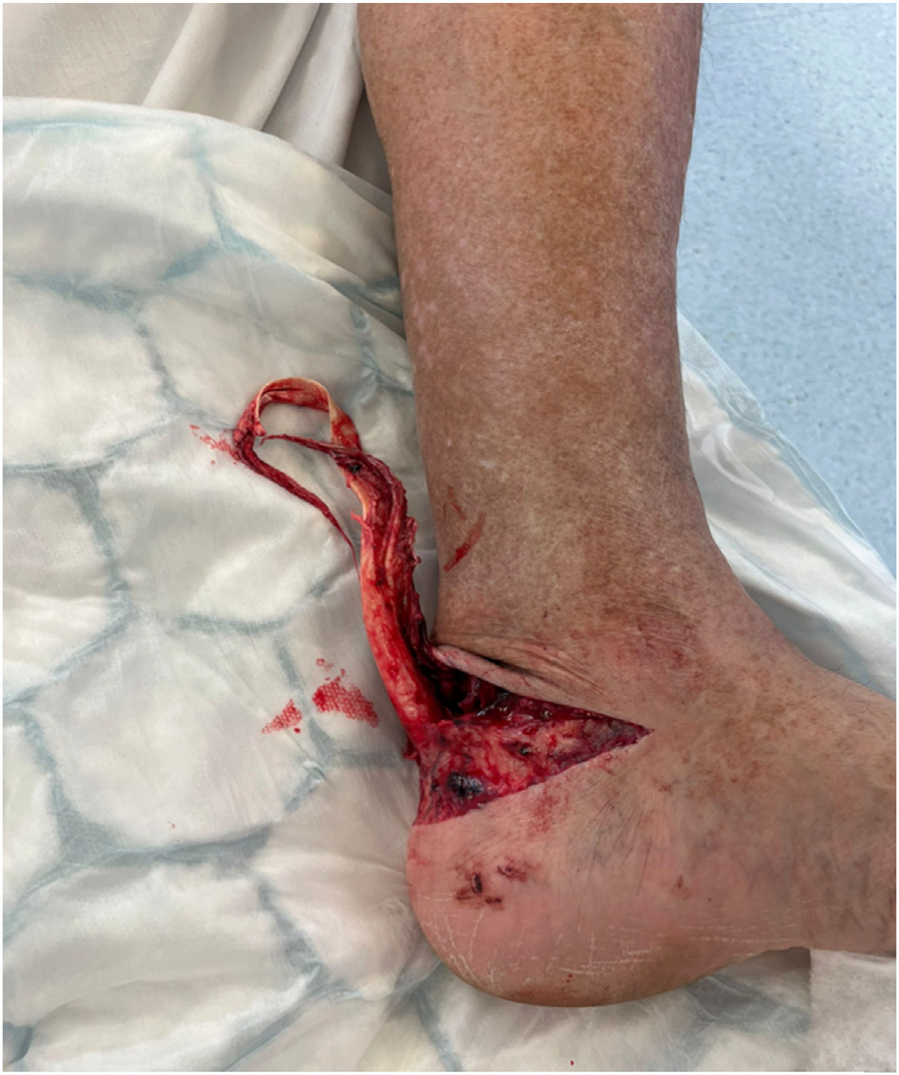
Open wound at time of injury.

He reported descending a set of stairs backward, when his left foot got caught in between two steps resulting in forced dorsiflexion and fall three metres down the stairs. This resulted in an open wound to the posterior aspect of the left heel, extending from the medial to lateral malleolus (Fig. 1). Initial primary and secondary surveys were otherwise unremarkable with this being an isolated injury to the left ankle.

His co-morbidities included type 2 diabetes mellitus (managed with oral therapy), hypertension, two previous strokes, hypercholesterolaemia, gout and obesity. He was previously independent with all activities of daily living, a non-smoker, and independently mobile. Although no fracture was present, the patient's initial treatment was in line with British Orthopaedic Standards for Trauma (BOAST) for Open Lower Limb Fractures^4^ and he was given a dose of IV Co-Amoxiclav, and, after being photographed, the wound covered in saline-soaked gauze with an overlying occlusive film.

The patient underwent a joint Orthoplastics procedure in which an attempt was made to primarily re-attach the avulsed tendon to the muscle belly. This was then covered with a concurrent lateral rotational flap. Ten days later, the distal aspect of the flap was found to be necrotic with eschar. This warranted further washout and debridement of the wound. The defect was left open and managed with vacuum-assisted closure (VAC) therapy. A provisional plan was made to close the defect with a free flap at a later date.

Six days later, the patient was discussed in the Orthoplastic multidisciplinary team (MDT) meeting, where it was decided to excise the non-viable tendon to allow coverage of the defect with a SSG. This was closely monitored by the Plastics team. A week later, after further input from the Orthoplastic limb reconstruction MDT team, this gentleman went on to have an anterolateral thigh (ALT) flap. It was decided that a flexor hallucis longus (FHL) tendon transfer could be considered later if functionally required. The patient was followed-up in the outpatient Plastics and Orthoplastic outpatient clinic for monitoring of graft. Graft take was over 95%. However, assessment at the Orthopaedic clinic found that his left foot was fixed in 30° dorsiflexion. Efforts to correct this resulted in involuntary firing of the unopposed tibialis anterior. The patient will therefore require further operative intervention to correct this deformity and permit ambulation. This is likely to take the form of an arthroscopic ankle fusion rather than an FHL tendon transfer and ALT flap given prior flap failure and successful take of the SSG.

## Discussion

Hypovascularity of the tendon mid-substance can account for the majority of ruptures occurring at this site. Reduced vascularity and subsequent reduction in tensile strength has been reported at the site of tendon origin and insertion. This may account for the reported occurrence (although less commonly) of rupture at these sites.^5^ However, following an extensive PubMed/Medline search, we were unable to find any reports of a complete avulsion of the tendon from the gastrocnemius-soleus complex.

The decision to attempt primary repair with concurrent flap (and subsequent failure) resulted in three operations, with a fourth expected imminently. Although typically a salvage procedure, an FHL tendon transfer with concurrent rotational flap may have been performed from the outset. A similar one-stage repair has been reported by Nazerali et al. (2013), with excellent functional outcomes.^6^ The literature indicates an increased likelihood of suboptimal outcomes for our multimorbid patients^7^ as well as high rates of failure when attempting a muscle-tendon repair,^8^ as in this case. Another possible alternative would have been to attempt tendon debridement and primary closure followed by a delayed FHL tendon transfer after settling of the initial insult.

In the absence of literature studying this rare injury variant, it's difficult to account for it. Once could posit that it can be explained by the unusual mechanism of injury, with the patients’ foot getting stuck resulting in an angle of forced dorsiflexion greater than typically seen in a sporting-related rupture.

## Conclusion

We report a unique variant of ATR and discuss its cross-speciality management. Primary repair may not always be the best course of action, particularly for unusual injuries and multimorbid patients, as reported here. Discussions between specialists at time of injury can avoid multiple procedures to achieve the functional goal and avoid prolonged hospital stays.

## Declaration of Competing Interest

None.
